# Endothelial progenitor cells in ischemic stroke: an exploration from hypothesis to therapy

**DOI:** 10.1186/s13045-015-0130-8

**Published:** 2015-04-11

**Authors:** Ya-Feng Li, Li-Na Ren, Geng Guo, Lee Anne Cannella, Valeria Chernaya, Sonia Samuel, Su-Xuan Liu, Hong Wang, Xiao-Feng Yang

**Affiliations:** Centers for Metabolic Disease Research, Cardiovascular Research, and Thrombosis Research, Department of Pharmacology, Temple University School of Medicine, Philadelphia, PA 19140 USA; Department of Nephrology and Hemodialysis Center, The Second Hospital, Shanxi Medical University, Taiyuan, Shanxi Province 030001 China; The First Clinical Medical College, Shanxi Medical University, Taiyuan, Shanxi Province 030001 China; Department of Neurosurgery, The First Hospital, Shanxi Medical University, Taiyuan, Shanxi Province 030001 China; Department of Biology, College of Science and Technology, Temple University, 1801 N. Broad St., Philadelphia, PA 19122 USA

**Keywords:** Ischemic stroke, Endothelial progenitor cells, Basic characteristics, Influence factors, Function, Therapy, Inflammatory factors, Review

## Abstract

As the population ages and lifestyles change in concordance, the number of patients suffering from ischemic stroke and its associated disabilities is increasing. Studies on determining the relationship between endothelial progenitor cells (EPCs) and ischemic stroke have become a new hot spot and have reported that EPCs may protect the brain against ischemic injury, promote neurovascular repair, and improve long-term neurobehavioral outcomes. More importantly, they introduce a new perspective for prognosis assessment and therapy of ischemic stroke. However, EPCs’ origin, function, influence factors, injury repair mechanisms, and cell-based therapy strategies remain controversial. Particularly, research conducted to date has less clinical studies than pre-clinical experiments on animals. In this review, we summarized and analyzed the current understanding of basic characteristics, influence factors, functions, therapeutic strategies, and disadvantages of EPCs as well as the regulation of inflammatory factors involved in the function and survival of EPCs after ischemic stroke. Identifying potential therapeutic effects of EPCs in ischemic stroke will be a challenging but an incredibly important breakthrough in neurology, which may bring promise for patients with ischemic stroke.

## Introduction

Ischemic stroke, also known as cerebral infarction, refers to a local blood supply obstacle that leads to cerebral anoxic lesions and ischemic necrosis, which subsequently causes a loss of corresponding neurological functions. Although few effective therapies are available, it is exceptionally crucial that patients who experience the onset of an ischemic stroke take drugs that have been implicated in providing effective cerebral protection and repairing the blood supply of ischemic penumbra as soon as possible. As the precursor cells of endothelial cells (ECs), endothelial progenitor cells (EPCs) have the characteristic of mobilization and can further proliferate and differentiate into mature ECs. Under the stimulus of physiological or pathological factors, bone marrow-derived EPCs can migrate to the peripheral blood where they participate in the repair of damaged blood vessels and angiogenesis in ischemic tissues. Recent studies on EPCs provide novel and promising potential therapies for the treatment of ischemic stroke and prognosis assessment improvement. In the following overview, we will elaborate regarding the role of EPCs in ischemic stroke.

### Basic characteristics of EPCs in ischemic stroke

#### Source and measurement

Niches, or specialized microenvironments that support the function of a specific cell, vary between cell phenotypes. Specifically for stem cells, niches provide a protected compartment shielded from toxic agents where stem cells can maintain their integrity and stemness but where certain stimuli can initiate proliferation. After ischemic stroke, stem cells and EPCs are stimulated and become mobilized in order to migrate from those niches. Since the initially recognized niche of EPCs is the bone marrow, many reports support that, after cerebral infarction, bone marrow-derived cells are a source of newly generated ECs [1]. On the other hand, increasing evidence indicates that EPCs can also be isolated and identified in both peripheral blood and human cord blood [2-4]. Interestingly, recent studies support that EPCs may particularly reside in the adult vascular wall and be able to differentiate into mature ECs [5], while c-kit + CD45− progenitor cells derived from the liver can migrate to the peripheral blood to supplement circulating EPCs [6]. This finding not only strengthens our perception of the origin of EPCs but also favors a shift away from the antiquated paradigm that vasculogenesis occurs only during the process of embryonic development. Additionally, EPCs can be cultured *in vitro* from mononuclear cells isolated from peripheral blood or bone marrow [7]. EPCs obtained from several of the sources described above may be used to promote recovery after ischemic stroke in the future [1].

To measure circulating EPCs, they should be isolated, identified, and quantified. As our previous studies demonstrate, EPC populations can be better defined by analyzing the surface markers expressed on the cells such as CD34, CD133, and vascular endothelial growth factor receptor-2 (VEGFR-2, KDR) using flow cytometry; after that, the cells have been positively stained. Additionally, functional EPCs can also be isolated by utilizing a colony-forming unit (CFU)-Hill, an endothelial colony-forming cells (ECFCs) assay, or cell adhesion to fibronectin-coated dishes with specific lectin and lipoprotein binding properties [8]. Nonetheless, the methodology of cell culture assays remains popular. In the course of this approach, EPCs remain quiescent during the early stages of culture (within 48 h) but are quickly stimulated to proliferate and differentiate into late EPCs or developed ECs (2 weeks), ultimately generating cell products that can be analyzed based on count and morphology [9]. Considering that the markers of the progeny of ECFCs and ECs are nearly indistinguishable, we can further enrich the population of ECFCs’ progeny by gating out monocytes, red blood cells, dead cells, and CD45+ blood cells [10].

### Mobilization, migration, and differentiation

The ability of EPCs to repair ischemic injuries requires them to first be mobilized in order for them to be able to migrate into the ischemic region, where the EPCs can then differentiate into mature ECs. These processes are mediated mainly via growth factors which include, but are not limited to, stromal-derived factor (SDF-1), VEGF, granulocyte-colony-stimulating factor (G-CSF), stem cell factor, soluble intercellular adhesion molecule, granulocyte-monocyte-colony-stimulating factor, hepatocyte growth factor, interleukin-6 (IL-6), IL-10, estrogen, and endothelial nitric oxide synthase (eNOS) [11]. Additionally, severe forms of chronic brain hypoperfusion in intracranial atherosclerotic disease (ICAD) might further stimulate the mobilization of EPCs and angiogenic growth factor (AGF) production [12].

**Figure 1:**
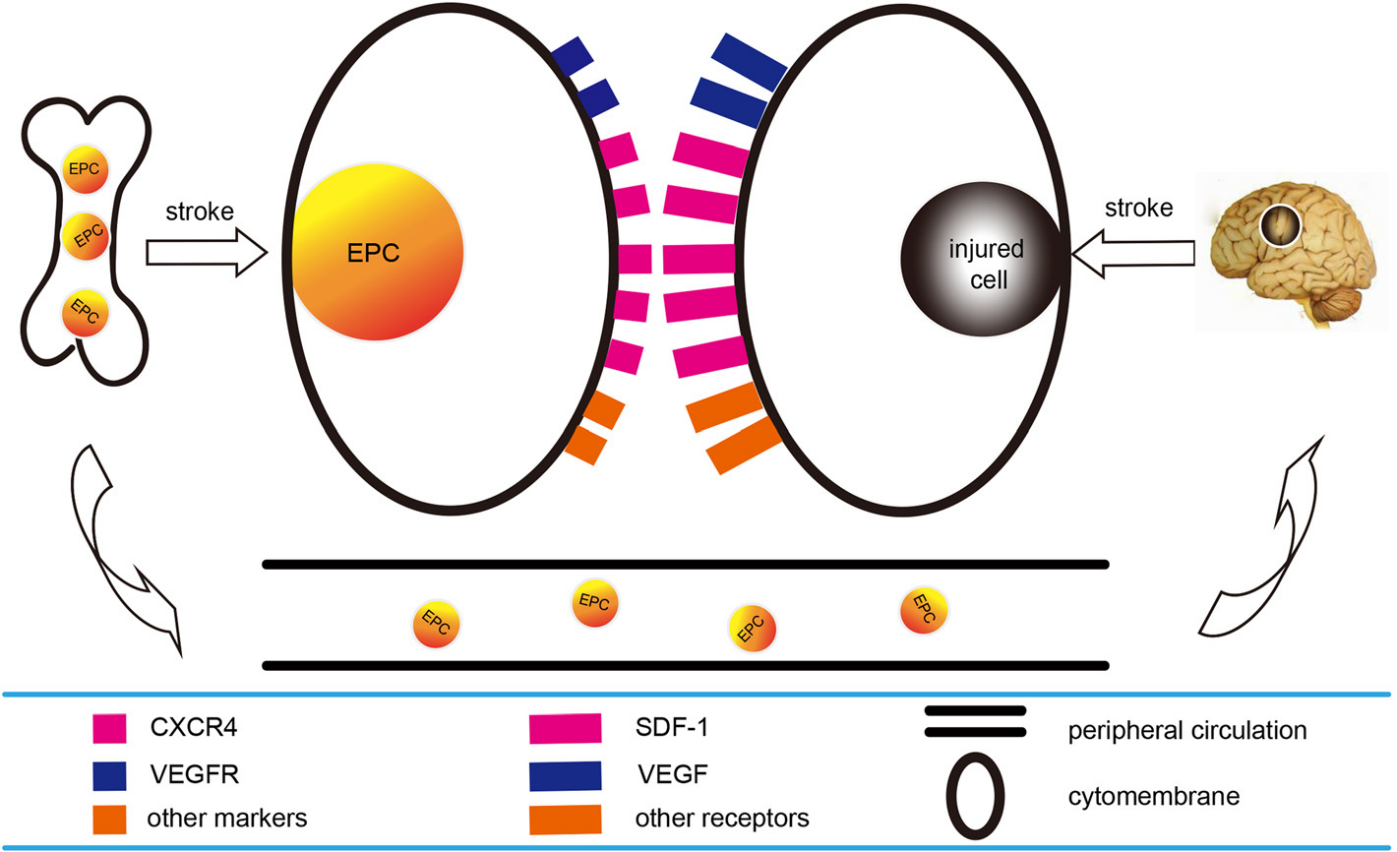
SDF-1/CXCR4 and other factors work together to bring more EPCs to ischemic brain regions.

**Figure 2:**
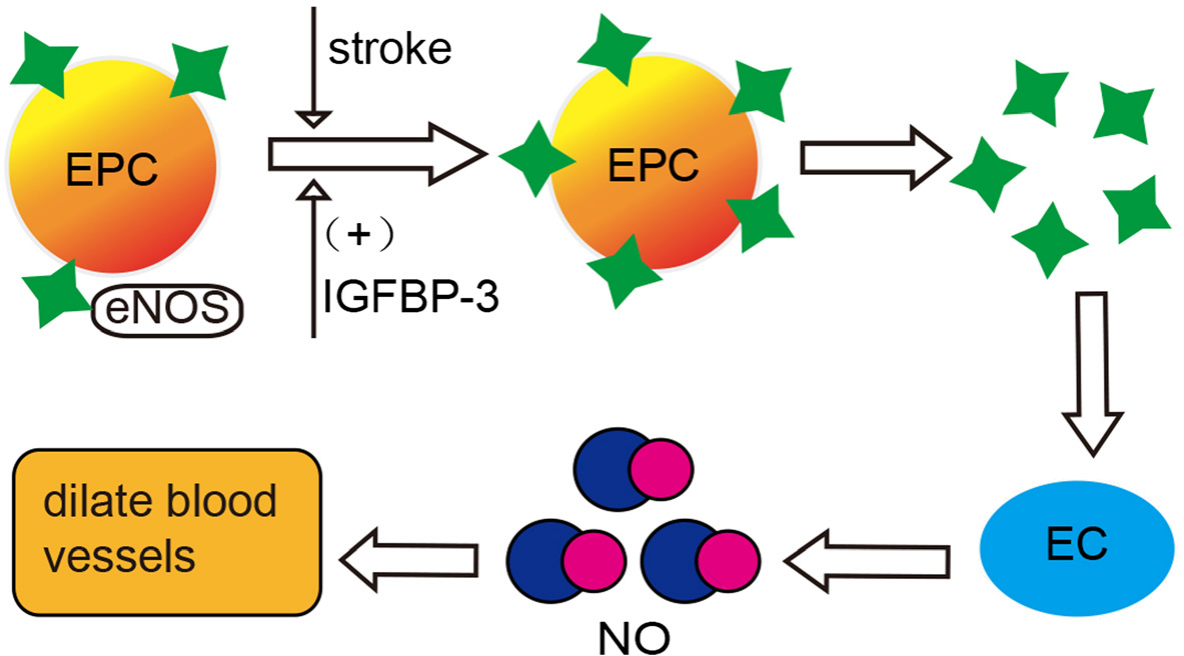
EPCs use IGFBP-3/eNOS/NO pathway to improve vessel dilation and blood supply to the ischemic areas.

The processes of EPC mobilization and migration are influenced by several signal transduction pathways in the cells. As shown in Figure 1, the first pathway, involving SDF-1 and C-X-C chemokine receptor type 4 (CXCR4)-mediated signaling [13-15], depends on the binding of a ligand to its receptor. The majority of EPCs express CXCR4. After ischemic stroke, SDF-1 expression in the injured brain is remarkably up-regulated, while CXCR4 expression on EPCs is enhanced. The resulting increased amount of SDF-1 in the brain attracts additional EPCs expressing CXCR4 to the ischemic region. Subsequently, EPCs can then implement functions to augment the repair of injured ECs, blood vessels, and even nerves. The mediation of SDF-1/CXCR4 to EPCs also cooperates with other growth factors such as VEGF/VEGFR [11], KDR/CD34 [16], and G-CSF/stem cell factor (SCF) [17] and so on. Figure 2 shows the second pathway: eNOS-dependent signaling. It has been reported that most EPCs can express eNOS, and that expression is increased following ischemic stroke [18]. Up-regulated eNOS then promotes EPCs to move to ischemic sites and subsequently raises the levels of endogenous nitric oxide (NO) in the brain which can dilate blood vessels, relax vascular smooth muscle cells, increase blood flow, promote blood circulation, and regulate blood pressure and blood fat, ultimately inhibiting atherosclerosis. Interestingly, a report showed that insulin-like growth factor binding protein (IGFBP-3) could increase the expression of eNOS, which may contribute to the activation of high-density lipoprotein receptor and the phosphatidylinositol 3-kinase/Akt pathway [19]. Moreover, estrogens mobilize EPCs through an eNOS-mediated mechanism as well as through direct actions on the alpha and beta estrogen receptors via matrix metalloproteinase-9 (MMP-9) [8]. Taken together, this data indicates that eNOS-dependent signaling also influences the migration of EPCs.

Furthermore, insulin-like growth factor 2/mannose-6 phosphate (IGF2/M6P) receptor (IGF2R) is highly expressed in EPCs, but its ligand, IGF2, which is a hypoxia-inducible gene, is absent in the normoxic condition. However, cells under hypoxic stress secrete IGF2 and subsequently induce EPC chemotaxis through binding with IGF2R, which can be inhibited by mannose-6-phosphate. The effect of IGF2 on EPC chemotaxis is much stronger than VEGF, which makes IGF2 similar to SDF-1. In addition, IGF1 has an effect on EPC chemotaxis similar to but relatively lower than that of IGF2 [20]. A study has shown that IL-10 knockout (KO) mice reduce myocardial-infarction (MI)-induced mobilization of bone marrow EPCs, down-regulate the expression of CXCR4 and VEGF, and enhance the susceptibility to inflammation or hypoxia-induced apoptosis, which can be reversed by systemic IL-10 therapy [21]. In addition, hypoxia-inducible factor (HIF)-1alpha regulates the expression of VEGF, SDF-1, and CXCR4, which can be up-regulated by cobalt and hydralazine. Stabilizing HIF-1alpha protein enhances EPC mobilization and function [22]. EPC migration and adhesion can also be regulated by angiopoietin-2 (Ang-2), a soluble ligand on ECs at the sites of vessel remodeling and angiogenesis. Ang-2 causes a remarkable stimulation of EPC migration, which can be inhibited by soluble Tie2 (a receptor tyrosine kinase of the Tie family) ectodomain, which binds Ang-2 as its ligand [23]. Similarly, thrombin and its receptor, protease activated receptor-1 (PAR-1), could promote the mobilization, proliferation, and differentiation of EPCs [24]. Another study showed that retinal cells release neurotrophic factors under hypoxic conditions to enhance EPC activity *in vitro* and increase angiogenesis in a mouse ischemic hind-limb model [25], but whether they have the same role on EPCs after ischemic stroke is not yet established.

With regard to the differentiation of EPCs, except for the above involved factors, VEGF can enhance the effects on the differentiation from EPCs into mature endothelium, which can be significantly promoted by fibronectin via specific synergism with alpha-5-beta-1 integrin [26]. Bone morphogenic protein 4 (BMP4) is a specific marker of late EPCs and plays a key role in EPCs’ commitment and outgrowth during neovascularization [27]. It has been well established that cyclic-adenosine monophosphate (cAMP) is an important secondary messenger in mediating many physiological functions including cell differentiation. It has been reported that in the mouse embryonic stem cells system, 8-bromoadenosine 3′, 5′-cAMP(8-Br-cAMP, a cell-permeable cAMP analog) can promote arterial specification of ECs [28]. A report indicated that mitogen-activated protein kinase kinase (MEK, MAPKK)/extracellular signal-regulated kinase (ERK) can modulate the fibrous tissue remodeling in the periodontal ligament (PDL) by affecting the proliferation, migration, and differentiation of the PDL-derived EPC-like cells [29]. In a similar fashion, the issue of whether this signal exists in the differentiation of EPCs after ischemic stroke is not fully understood.

In conclusion, although the abovementioned growth factors have been studied extensively, a more exact mechanism still remains to be determined.

### Homing

#### Pathways of homing

**Figure 3:**
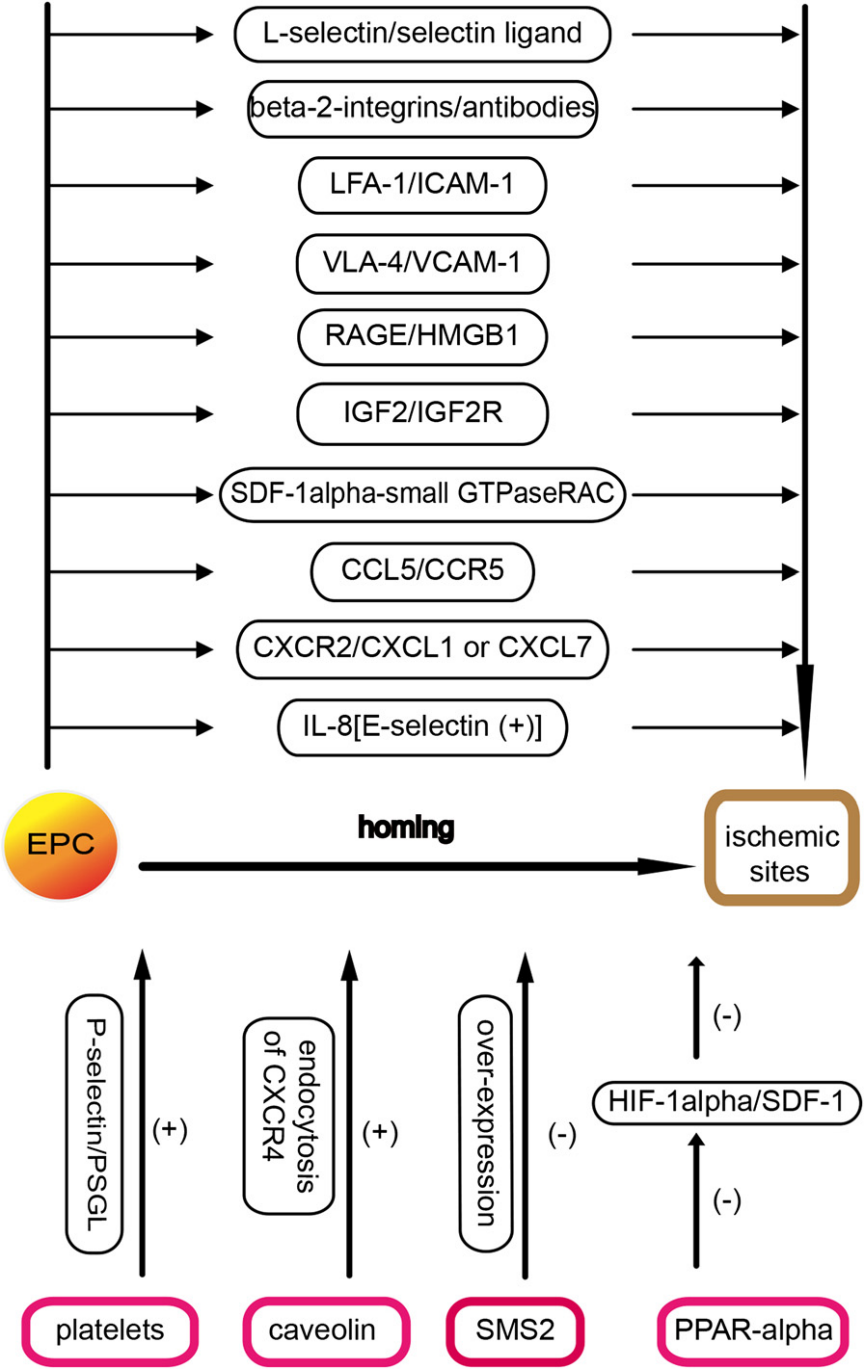
Multiple pathways regulate EPCs homing to the ischemic sites.

After ischemic stroke, the homing of EPCs to the neovascular zone has been considered an essential step in the formation of vascular networks, which involves the interaction of many cytokines and their receptors. Some specific pathways are as follows (Figure 3).

To illustrate one pathway, Biancone et al. [30] showed that the homing of EPCs involves the expression of L-selectin, an adhesion receptor on EPCs, and its ligand in ischemic sites. Chavakis et al. [31] demonstrated in a murine model of hind-limb ischemia that preactivation of the beta-2 integrins expressed on EPCs via activating antibodies enhances the homing and neovascularization of EPCs *in vivo*. Moreover, Duan et al. [32] indicated that leukocyte function-associated antigen (LFA-1, alpha-L-beta-2, and CD11a/CD18) and integrin alpha-4-beta-1 (very late antigen-4, VLA-4) expressed in human umbilical cord blood-derived high proliferative potential-endothelial progenitor cells (HPP-EPCs) are involved in HPP-EPCs homing to ischemic tissues via the interaction with their ligands, intercellular adhesion molecule-1 (ICAM-1) and vascular cell adhesion molecule-1 (VCAM-1), expressed in vessel endothelium in ischemic tissues, which can be blocked by CD11a and CD49d antibodies. Lev et al. [33] demonstrated that platelets play a role in the homing of EPCs to injury sites. The interaction between EPCs and activated platelets under static and flow conditions is mediated through P-selectin-P-selectin glycoprotein ligand-1 (PSGL-1) interaction *in vitro*, which is inhibited by antibodies to P-selectin or PSGL-1, but not by antibodies to glycoproteins Ib-IX-V or IIb/IIIa. Additionally, Sbaa et al. [34] reported that EPC transduction with caveolin small interfering RNA, which mediates the endocytosis of CXCR4, regulating both the SDF-1-mediated mobilization and peripheral homing of EPCs to ischemia sites, leads to a more extensive rescue of the ischemic hind-limb. Chavakis et al. [35] showed that EPCs express high-mobility group protein B1 (HMGB1) receptors, including receptors for advanced glycation end products (RAGE) and toll-like receptor 2 (TLR2), but HMGB1 stimulates the migration of EPCs in a RAGE-dependent manner, where HMGB1 increases EPC adhesion to the immobilized integrin ligands, ICAM-1 and fibronectin. Besides, HMGB1 rapidly increases integrin affinity and induces integrin polarization. What is more, Maeng et al. [20] reported that the remarkably promoting role of the IGF2/IGF2R system in EPC homing is mainly via IGF2R-linked G(i) protein signaling and intracellular calcium (Ca^2+^) mobilization which is induced by the beta-2 isoform of phospholipase C. Zhao et al. [36] thought that sphingomyelin synthase 2 (SMS2) over-expression is probably associated with an increase in expression of aortic inflammatory biomarkers, as well as a decrease in the number of CD34/KDR-positive cells, circulating angiogenic cells (CACs), and CFU in circulation in apolipoprotein E (ApoE) KO mice. What is more, Shen et al. [37] indicated that the SDF-1alpha-small GTPaseRAC signaling pathway plays an important role in polarity, morphology variation, and the direction of migration of EPCs, which is induced by SDF-1alpha. Finally, Wang et al. [38] showed that peroxisome proliferator-activated receptor (PPAR)-alpha restrains ischemia-induced EPC mobilization and homing via inhibition of the HIF-1alpha/SDF-1 pathway during retinal neovascularization. Furthermore, chemokine (C-C motif) ligand 5 (CCL5) induces *in vitro* EPC homing in a C-C chemokine receptor type 5 (CCR5)-dependent manner. The CCL5/CCR5 interaction will be a novel molecular target for modulation of neovascularization and eventual tissue repair [39]. EPCs can express C-X-C chemokine receptor type 2 (CXCR2), and damaged arterial smooth muscle cells (SMCs) up-regulate the expression of chemokine (C-X-C motif) ligand 7 (CXCL7) and CXCL1. The binding between CXCR2 and CXCL1 or CXCL7 can also enhance the homing of circulating EPCs to sites of arterial injury and endothelial recovery *in vivo* [40]. E-selectin not only stimulates ECs to express ICAM-1 but also EPCs to secrete IL-8, leading to enhanced homing and incorporation of EPCs to ECs capillary formation [41]; the issue of whether this process occurs after ischemic stroke remains to be distinctly elucidated.

#### Enhancing homing efficiency

As mentioned above, EPC homing is crucial and poor homing efficiency is one of the major limitations of current EPC therapies. Hence, the issue of how to enhance EPC homing becomes particularly important. In this aspect, the following addresses that recent research has begun to make progress in EPC homing efficiency.

Wang et al. [42] found that one of the effects of angiotensin-converting enzyme inhibitor, enalapril, on the cardiovascular system involves the modulation of levels of circulating EPCs by the CD26/dipeptidyl peptidase IV (DPP-IV) system. In the blood, through the anti-inflammatory effect, enalapril significantly decreases CD26+ cell numbers then leads to a decrease in total DPP-IV activity. However, in the bone marrow, enalapril does not influence CD26+ cell numbers, but it does enhance DPP-IV activity. Evaluated results about a novel strategy using magnetic bionanoparticles (MPs) to enhance the homing of transplanted EPCs to ischemic tissue showed that below threshold concentration, MP transfer can enhance the migration and homing without influencing proliferation or survival of EPCs, in which the effect of high dose MP is better than that of low dose MP [43]. Georgescu et al. [44] have shown that the treatment with angiotensin II receptor antagonist, irbesartan, significantly increases EPC infiltration and decreases MP infiltration by diminishing pro-inflammatory endothelial cytokines such as VEGFR-1, VEGFR-2, CXCR4, Tie2, and placental growth factor with role in EPC homing to sites of damaged endothelium, as well as by increasing protein expression of cyclooxygenase 2 and prostacyclin synthase molecules with a role in the improvement of arterial wall vasodilatation.

In brief, although the roles of these cytokines and their ligands on the homing of EPCs has been indicated by many studies, it is still important to further improve our understanding on the exact mechanism regarding the homing of EPCs in order to enhance EPC homing efficiency following ischemic stroke.

### Immune phenotypes

Many studies have indicated that circulating EPCs cannot only express human stem cell markers such as CD34, CD45, CD62, or CD133 but can also express endothelial cell markers such as CXCR4, CD31, VEGFR-2 (KDR or Flk1), Von Willebrand factor (vWF), vascular endothelial cell-Cadherin, Tie2, or eNOS [18], which participate in maintaining the vascular homeostasis by monitoring and repairing dysfunctional endothelium [16,45-47]. Moreover, it has been reported that Flk1/CD34-double-positive EPCs can express pro-recovery mediators such as the brain-derived neurotrophic factor and basic fibroblast growth factor [48]. Additionally, Brea et al. [49] indicated that reticulum protein-29 and cell division control protein 42 homolog are only expressed in EPCs from healthy subjects, whereas elongation factor-2 is only identified in EPCs from ischemic stroke patients. Currently, more studies investigating immune phenotypes of EPCs are under way.

### Time distribution

Identifying the specific time distribution of EPCs after ischemic stroke will guide us to conduct assessments of EPC therapy. Firstly, in animal experiments, a report using rats showed that EPCs are transplanted after a 90-min middle cerebral artery occlusion (MCAO), and myeloperoxidase-immunoreactive cell counts and neurological deficit scores are reduced after 24 h; the result presents an increase in regional cortical blood flow and a decrease in infarct volume after 48 h [18]. Secondly, the study about patients with first episode of nonlacunar ischemic stroke has reported that serum levels of VEGF at 72 h and SDF-1alpha levels at 24 h are independent factors of EPC increase during the first week of evolution of the disease [50]. Furthermore, in clinical trials, Zhou et al. [51] showed that in Chinese patients with acute stroke, EPCs gradually increase by day 7 after acute onset, remain elevated by day 14, and return to baseline by day 28. It is valuable for us to determine the severity of human EPC changes following ischemic stroke as time advances.

### Influence factors of EPCs in ischemic stroke

It is well known that many factors contribute to the occurrence of cerebrovascular diseases such as high blood pressure, heart disease, diabetes, dyslipidemia/hyperlipidemia, cigarette smoking, alcohol, elevated body weight, carotid stenosis, hyperhomocysteinemia/high homocysteine, high fibrin, lack of physical exercise, unbalanced diet, and so on. Recently, more and more studies have begun paying closer attention than previous reports had to these risk factors as well as the correlation of EPCs in ischemic stroke.

### Negative factors

Atherosclerosis is one of the conventional risk factors of cerebrovascular diseases, mainly on account of the vascular stenosis caused directly by ruptured atherosclerosis and the embolism of distant small blood vessels due to plaque falling off. Intracranial atherosclerotic disease is an important cause of ischemic stroke, and endothelial dysfunction plays a critical role in its onset and progression [12]. In ApoE-deficient mice, repairing functions of EPCs are weakened, which is correlated with atherosclerosis caused by the chronic stimulation of hyperlipidemia. C-reactive protein (CRP) causes a concentration-dependent increase in reactive oxygen species (ROS) and apoptosis, as well as a decrease in glutathione peroxidase. Oxidized low-density lipoprotein induces apoptosis and senescence of EPCs. Carbamylated low-density lipoprotein induces an increase in oxidative stress and mitochondrial depolarization and a decrease in EPC proliferation and angiogenesis [8]. All aspects mentioned above participate in the development of atherosclerosis, which also suggests that the reduction of circulating EPCs may contribute to the development of atherosclerosis and, in turn, enhancing EPCs may reduce the injury of atherosclerosis by protecting and repairing the damaged vascular functions [52]. Moreover, a high Hcy level in peripheral blood, as an independent risk factor, leads to vascular injury independently or synergistically with other risk factors, which can contribute to atherosclerosis and then cause an ischemia. It has been observed that there is an inverse correlation between EPC levels and Hcy levels, which can be reversed by B vitamin intervention. Mediated by high Hcy, EPC toxicity mainly involves apoptosis that may refer to the activation of caspase-8 and caspase-9, cytochrome C release, and caspase-3 activation [53]. Endothelin (ET) has been found so far to be the strongest substance involving the shrinkage of blood vessels. It can cause vasoconstriction, myocardial ischemia, metabolic disorders, and cell proliferation, which are all common risk factors of vascular injury-related diseases including ischemic stroke. ECs have been shown to reflect the degree of endothelial damage, as they may be responsible for increased ET-1 expression, which may further play a role in the pathophysiology of stroke and subsequent EPC mobilization [54]. It has been reported that circulating EPCs are inversely correlated with parameters such as blood glucose levels and ischemic damage, except for cerebral microvascular density in diabetic stroke. In addition, cellular membrane microparticles impair EPCs’ functions [55]. According to the report, we can postulate that increasing EPCs’ generation, improving EPCs’ function, and reducing production of cellular membrane microparticles may attenuate ischemic damage. In early ischemic stroke, EPCs and markers of neuroinflammation that include up-regulated adhesion molecules such as ICAM-1, VCAM-1, E-selectin, tumor necrosis factor (TNF)-alpha, IL-6, ET-1, and markers of tissue injury including MMP-9 and tissue inhibitor of matrix metalloproteinases-1 (TIMP-1) have a negative correlation [56]. Body mass index, blood pressure, LDL, total cholesterol, and high-sensitivity C-reactive protein also have an inverse correlation with EPC levels whereas systolic blood pressure and total cholesterol are independent predictors of EPC levels [51]. Tobacco smoking, high fat diet, hypertension, and hyperlipidemia remain as the major risk factors, and treatment of these conditions has been shown to significantly reduce the incidence of ischemic stroke [57].

### Positive factors

Hayakawa et al. [58] have shown us that reactive astrocytes could promote proliferation of EPCs and then improve neurovascular remodeling and functional recovery after ischemic stroke. An important molecular mechanism is as follows: reactive astrocytes release a damage-associated molecule known as HMGB1 and up-regulate HMGB1 in the peri-infarct cortex after ischemic stroke, which can raise EPC levels, and then increase peri-infarct angiogenesis to reduce the worsening of neurological deficits. Hayakawa et al. [48] further indicated that the pathway of astrocyte-EPC signaling might improve the recovery of white matter injury by enhancing the functions of EPCs such as migration, accumulation, and tube formation, which, however, is also dependent on HMGB1 from reactive astrocytes and its receptor RAGE. In addition, a recent study has shown that erythropoietin (EPO) therapy can astoundingly enhance the levels of circulating EPCs and improve 90-day major adverse neurological event (defined as recurrent stroke, National Institutes of Health Stroke scale is equal to or more than 8, or death) through a randomized clinical controlled trial [16,59]. As a subset of lymphocytes, angiogenic T-cells are able to stimulate EPC function [60]. Liu et al. [25] reported that neurotrophic factors may promote the activation and mobilization of EPCs along with subsequent neoangiogenesis. Moreover, IGFBP-3 prevented vascular endothelial cells and EPCs from undergoing apoptosis [19]. A report has shown that stem cells, including EPCs, from a variety of sources can be used as a tool to study and prevent the events that lead to ischemic stroke [57].

### Controversial factors

Angiotensin-converting enzyme 2 (ACE2) catalyzes the conversion of angiotensin I (Ang I) into Ang 1–9 and Ang II into Ang 1–7. It has been reported that Ang II down-regulates circulating EPC levels, impairs EPC function, and is detrimental to ischemic stroke *in vivo* and *in vitro* [61]. Furthermore, additional reports showed that ACE blockers and type 1 angiotensin receptor inhibitors protect EPC survival and functions [62,63]. However, Chen et al. [64] indicated that ACE2 could promote the migration of EPCs and tube formation, improve their function, and enhance the efficacy of EPC-based therapy for ischemic stroke based on the results from the *in vivo* and *in vitro* studies, which are mainly via regulating eNOS and nicotinamide adenine dinucleotide phosphate (NADPH) oxidase (NOX) pathways. Additionally, it has been put forward that ACE2 overexpression in blood vessels not only enhances the function of ECs [65] but also limits the early development of atherosclerotic injury by improving ECs’ function [66]. The previously stated relationship is further supported by a finding that ACE2 overexpression promotes the migration of EPCs and tube formation, which can be enhanced by NOX inhibitor and inhibited by ACE2 or eNOS inhibitor [64].

Taken together, EPC levels are inversely correlated with the various risk factors and positively correlated with astrocytes, EPO, and angiogenic T-cells, whereas the relationship of ACE2 and EPCs still remains controversial. In spite of the above ideas, by inhibiting negative factors and improving positive factors, it continues to be unclear whether or not EPCs play a positive role in ischemic stroke.

### Functions of EPCs in ischemic stroke

In recent years, EPCs have gained more and more attention, particularly regarding the relationship between EPCs and ischemic cerebral diseases. EPCs carry out basic activities within the vascular system such as paracrine signaling, healing of endothelial injury, and formation of new blood vessels in ischemic tissue. It has also been found that EPCs may play a critical role in the pathophysiology as well as tissue repair and regeneration in the ischemic brain [13,67].

### EPCs repair damaged ECs

**Figure 4:**
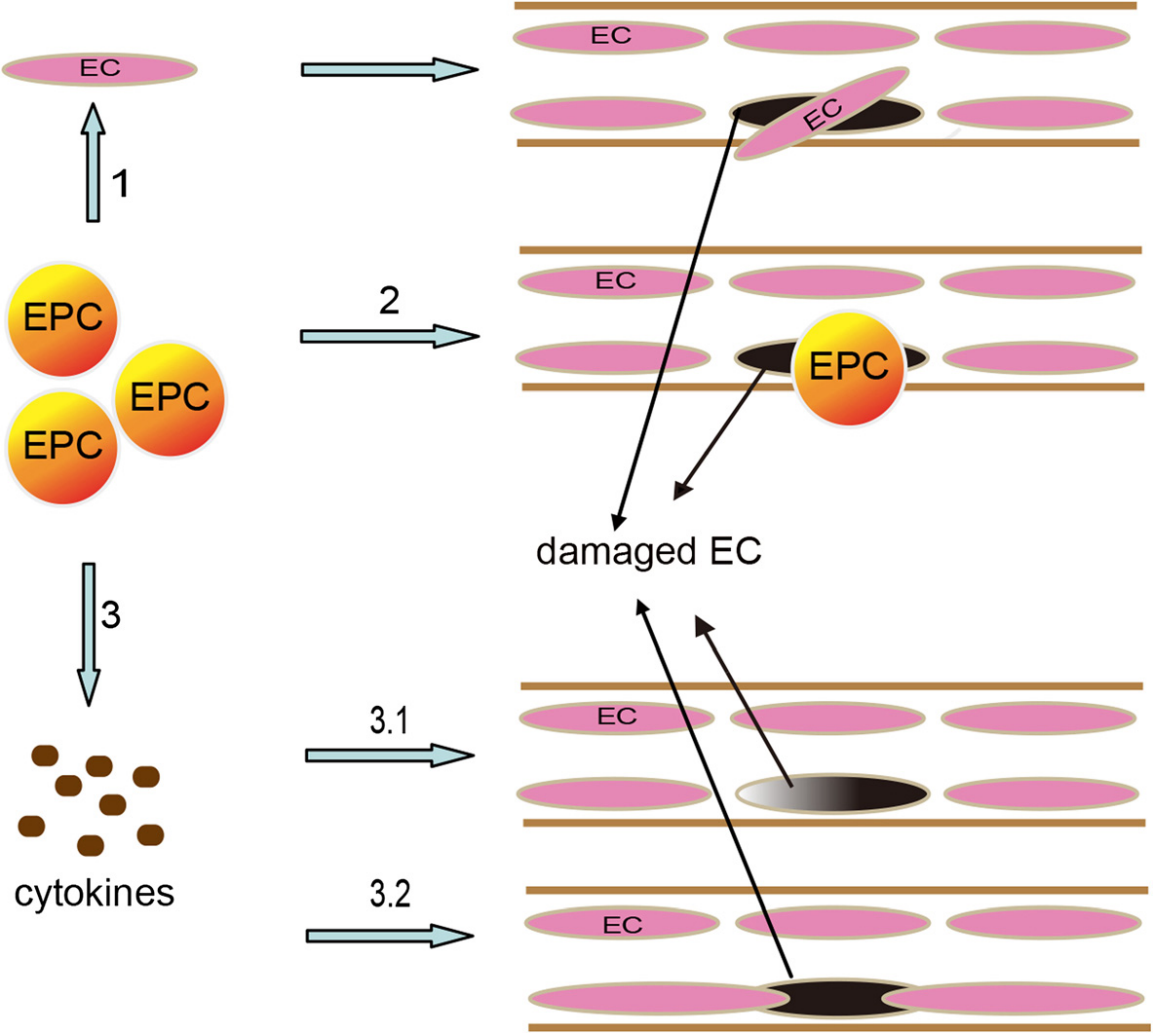
Mechanisms of EPCs repairing damaged ECs. 1. EPCs differentiate into mature ECs that repopulate injured sites; 2. Direct incorporation of EPCs; 3. EPCs can secrete protective cytokines that can promote self-repair of injured ECs (3.1) or mediate neighboring-injury ECs with normal structure and function to extend into injured sites and then perform the function of repair (3.2).

It is well known that a major pathophysiological event of ischemic stroke is vascular endothelial damage that is induced by various high-risk factors such as hypertension, hyperlipidemia, diabetes, and many more. Evidentially, the development of therapeutic approaches to repair damaged ECs has become an attractive topic of research. After ischemia, EPCs migrate from the bone marrow to the injured region in order to repair the damaged site either through direct incorporation of EPCs or by repopulating mature ECs [53]. Increasing numbers of investigators recognize that EPCs participate in endothelial repair with the following probable mechanisms: the first theory proposes that EPCs have an ability of differentiating into ECs, which can add to the levels of circulating ECs that may be transported to injured parts via blood flow to replace the injured ECs [7,68]. In controversy, the second theory proposes that EPCs can secrete protective cytokines and growth factors. On one side, these factors may promote self-repair of injured ECs, while on the other side, they may mediate neighboring-injured ECs with normal structure and function extending into injured sites and performing the function of repair [7,45,68]. The above viewpoints have been generally accepted, but the precise role of EPCs still remains unclear (Figure 4).

### EPCs promote the formation of new blood vessels

It has been reported that EPCs promote neurovascular remodeling and functional recovery after stroke and brain injury [58]. Several initial studies seem to support the concept that postnatal new vessels rely on angiogenesis or the process of forming new blood vessels from the existing capillaries or post-capillary venule [7,68]. As reports about EPCs gradually increase, researchers have come to realize that bone marrow-derived EPCs are not restricted to angiogenesis and are also involved in vasculogenesis after stroke [3]. Much like embryonic angiogenesis, vasculogenesis refers to the identification, mobilization, migration, adhesion, and endothelial differentiation of EPCs, and in particular, they may promote the formation of new vessels in sites without blood vessels such as areas of ischemic necrosis. Zhang et al. [69] obtained bone marrow-derived EPCs from transgenic mice that expressed beta-galactosidase regulated by an endothelial-specific Tie2 promoter and injected them into adult mice. The authors discovered enlarged and thin-walled blood vessels with sprouting or intussusception at the boundary of the ischemic injury, and that Tie2-lacZ-positive cells were incorporated into sites of neovascularization at the border of the infarct, which exhibited an endothelial antigenic marker known as vWF. In a way, these findings confirm the idea that EPCs promote the repair and regeneration of injured vessels simultaneously, which refers to the combined action of angiogenesis and vasculogenesis after ischemic stroke [70].

### EPCs promote injured nerve repair

**Figure 5:**
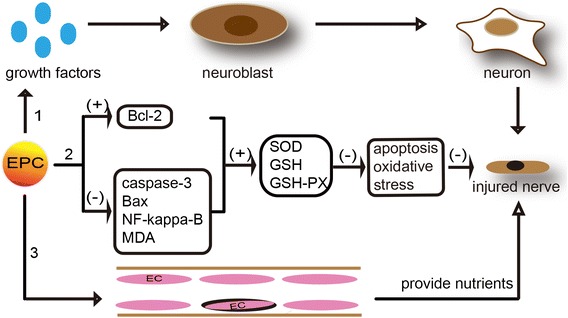
Mechanisms of EPCs promoting nerve repair.

Patients with ischemic stroke appear to have neuronal injury and necrosis, which give rise to changes in cognitive behavior. Promoting the repair and regeneration of injured nerves is also one of our therapeutic strategies. It is reported that 4 weeks after transient MCAO, the transplantation of EPCs reduces mouse cortex atrophy and improves neurobehavioral outcomes [13]. The specific mechanisms may correlate with the following: firstly, the activation of vessel growth factors results in neurogenesis and the migration of neuroblasts to the peri-ischemic cortex [71], which mainly refers to the proliferation and migration of subventricular zone neural progenitor cells along with a shift in cell fate to neurogenesis and oligodendrogliosis [72]. Additionally, EPCs may attenuate cell injury through various functions such as blocking neuronal apoptosis and oxidative stress. The detailed mechanisms involve up-regulating Bcl-2 expression, down-regulating expression of caspase-3, Bax, nuclear factor (NF)-kappa-B, and malondialdehyde (MDA) in the ischemic penumbra, which remarkably enhance activities of superoxide dismutase (SOD), glutathione (GSH), and glutathione peroxidase (GSH-PX) [73]. Furthermore, EPCs participate in endothelial repair and promote neurovascular formation, which could provide a good, nutrient-rich microenvironment for nerve repair, and reduce the cerebral infarct volume (Figure 5).

### EPCs evaluate the prognosis of ischemic stroke

It has been found that the levels of circulating EPCs are independently predictive of prognosis of ischemic stroke [74]. Following acute ischemic stroke, circulating EPCs are remarkably increased in order to execute their repair function on cerebrovascular trauma. Moreover, the increase of EPCs is associated with better functional outcome and a reduction of infarct growth [75]. However, low circulating EPC levels are independently predictive of severe neurological impairment measured at 48 h after ischemic stroke, but as time proceeds, major adverse clinical outcomes are fairly dynamic [74]. Furthermore, EPCs have different subsets such as the CFU and the population of outgrowth cells. We can evaluate the function of EPCs through the number and appearance of CFU, to then evaluate the prognosis of ischemic stroke [76]. Interestingly, a decrease of circulating EPCs with subsequently impaired EC repair can reduce arterial elasticity, which is a hallmark of aging in healthy humans.

Altogether, EPCs may protect the brain against ischemic injury, promote neurovascular repair, improve long-term neurobehavioral outcomes, and assist the evaluation of prognosis of acute ischemic stroke. However, it presently remains unclear, referring to the correlation between angiogenesis and neurogenesis, how to measure EPCs’ quantity, quality, and properties, and how to comprehensively evaluate the prognostic values of EPCs on ischemic stroke.

### Therapy

Considering the previously described functions of EPCs, EPC-based treatment of ischemic stroke has become a novel therapeutic strategy [2,77]. Importantly, the level of EPCs *in vivo* is slender and limited, comprising just less than 0.0001% to 0.01% of the peripheral circulating mononuclear cells, so the proper methods of administrating EPCs as a therapy are valuable to develop. Current researches have put forward a dichotomy of strategies which includes an expansion of bone marrow-derived EPCs *in vivo* and *in vitro* [18], however, although the former is optimal, administration of *in vitro*-expanded EPCs is more practical and feasible.

### In vivo

Consequently, the main aspects are as follows: in terms of physical therapy, it has been reported that electroacupuncture of the specific acupoints known as “Quchi” and “Zusanli” can up-regulate levels of EPCs from peripheral blood and bone marrow, serum total NOS activities, VEGF levels, blood CXCR4+ cells, as well as blood SDF-1alpha protein content in cerebral ischemia/reperfusion injury rats, which may help alleviate injury, promote neovascularization, and improve cerebral ischemia [78-80]. Moreover, voluntary physical activity improves long-term stroke outcome by eNOS-dependent mechanisms related to improving angiogenesis and cerebral blood flow [81]. In terms of drug therapy, firstly, circulating EPC levels may increase after statin treatment, which is most likely related to NO, although it is still unclear [82]. Secondly, B-type natriuretic peptide (BNP) is a neurohormonal peptide that applies to treat chronic heart failure patients. However, Shmilovich et al. [83] tested that by enhancing the number, proliferation, adhesion, and migration of EPCs, BNP could promote angiogenesis, therefore making its therapeutic effect on ischemic stroke promising. Thirdly, phosphodiesterase III inhibitors, such as cilostazol, can increase circulating EPCs, promote migration and adhesion of human umbilical cord blood-derived EPCs to a fibronectin-coated plate and ECs, and decrease small-dense LDL after cerebral ischemia, which may be associated with inducing integrin expression and activating protein kinase A (PKA) and multi-purpose cAMP targeted (Epac) signals [84,85]. Finally, based on a clinical controlled trial, Sobrino et al. [86] found that a single administration of cytidine diphosphate-choline (CDP)-choline and the co-administration of CDP-choline and tissue-plasminogen activator (rt-PA) both increase the levels of EPCs in acute ischemic stroke, where the impact of the former is miniscule, but the latter is remarkable. Therefore, it is worthy to distinguish which effect occurs as a result of CDP-choline alone versus the co-administration of CDP-choline and rt-PA.

### In vitro

The process of EPC expansion *in vitro* has many approaches and effects. For example, autologous EPCs collected via a multiplication culture *in vitro* can then be re-injected into the same animal. Transfusion of lentivirus-ACE2-primed EPCs reduces cerebral infarct volume and neurological deficits and increases cerebral microvascular density and angiogenesis [64]. Moubarik et al. [4] demonstrated that ECFCs injected 24 h after MCAO settle in the injured area and improve functional recovery. Another study has shown that during the acute phase of cerebral infarction in rats, EPCs with low aldehyde dehydrogenase activity (Alde-Low EPCs) can be up-regulated and migrate into the infarct region, accompanied with a decrease of infarct volume [14]. The transplantation of Alde-Low EPCs naturally provides a new strategy for patients that have suffered acute cerebral infarction. What is more, colony-outgrowth endothelial cells (OECs) from stroke patients are a subtype of EPCs which, at an early stage, present higher levels of pro-angiogenic factors such as CCL2, inhibitor of DNA binding 3, IGF-1, MMP9, transforming growth factor beta receptor 1, TNF-alpha-induced protein 2, TNF, and TGF-beta-1; whereas mature OECs present an increase in brain-specific angiogenesis inhibitor 1, neuropilin 2, thrombospondin 1, MMP2, and VEGF-C expression [87,88]. Despite changes in angiogenic-related gene expressions of OECs in the progress of expansion, the pro-angiogenic potential is always kept intact. Therefore, it would be beneficial to improve the cell-based therapy by intervening at the stage of EPC differentiation and to test autologous transplantation of EPCs using corresponding markers at different stages.

**Figure 6:**
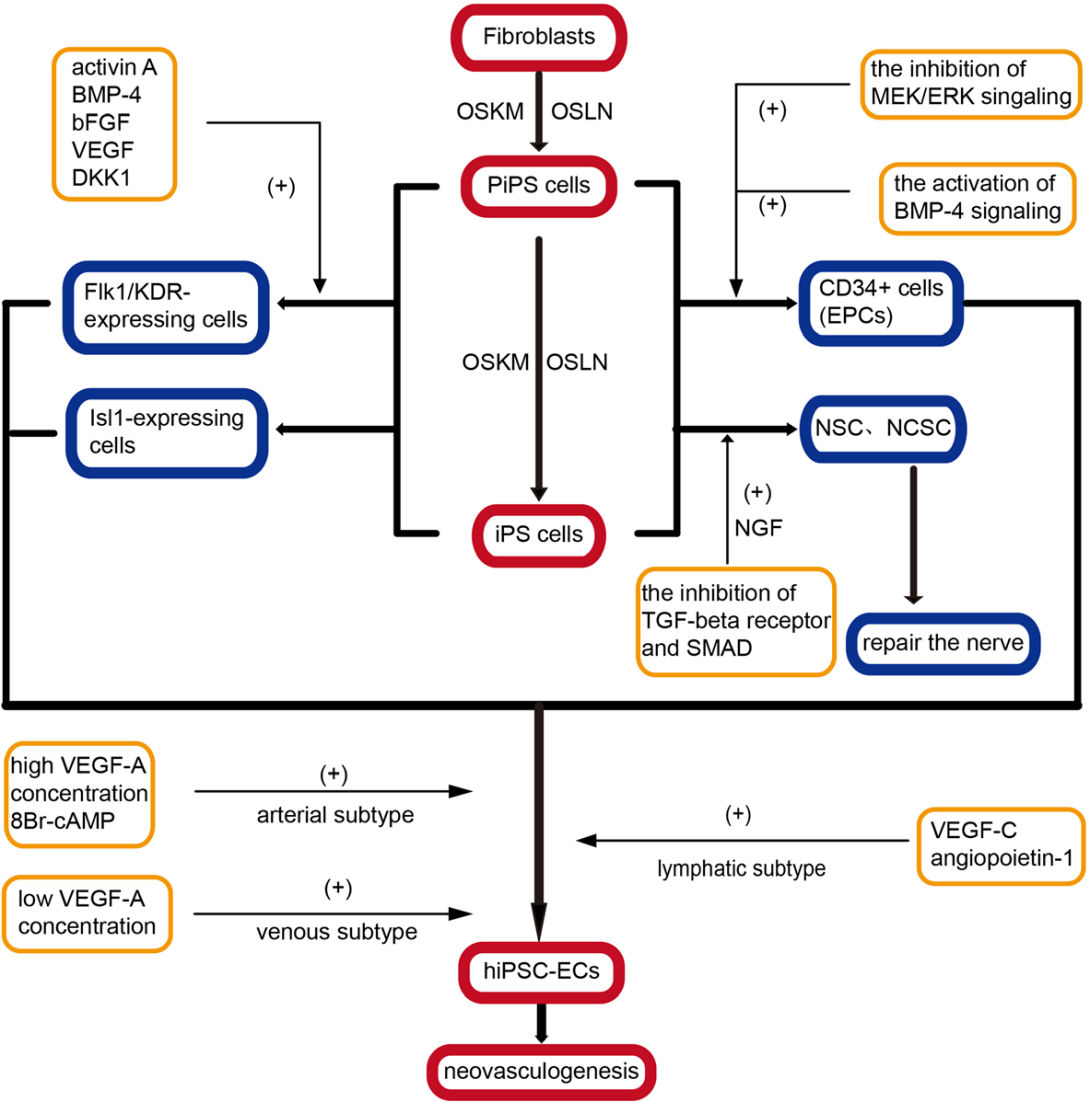
Induced pluripotent stem cells may be used as novel therapies for neovasculogenesis and ischemic stroke.

Moreover, recent studies about human-induced pluripotent stem cells (hiPSCs) put forward a new promise for the treatment of EPCs in ischemic stroke. One study in particular demonstrated that hiPSCs can differentiate into many cell types including endothelium, which can exhibit the rich functional phenotypic plasticity of mature primary vascular endothelium [89]. IPS cells are initially derived from fibroblasts and are then reprogrammed into partial iPS (PiPS) cells, and then into iPS cells using the transcription factors Oct4, Sox2, Klf4, and c-Myc (OSKM) or Oct4, Sox2, Lin28 and Nanog (OSLN). PiPS cells and iPS cells can differentiate into fetal liver kinase-1 (Flk1/KDR)-expressing cells and Islet-1 (Isl1)-expressing cells and then directed to ECs. PiPS cells can be enhanced by activin A, BMP-4, basic fibroblast growth factor, VEGF, and Dickkopf homolog 1. Worthily, CD34+ EPCs that can further differentiate into ECs to contribute to neovasculogenesis in ischemic regions could be derived from hiPSCs via the inhibition of MEK/ERK signaling and the activation of BMP-4 signaling. Moreover, iPS cells can differentiate into neural stem cells (NSCs) and neural crest stem cells (NCSCs) for neural tissue regeneration, improved by neural growth factor (NGF) and the inhibition of TGF-beta receptors and SMAD (mothers against decapentaplegic homologs, signal transducers, and transcriptional modulators that mediate multiple signaling pathways) signaling [90]. In addition, hiPSC-ECs purified based on positive expression of CD31 are heterogeneous in nature, displaying arterial, venous, or lymphatic subtypes, which are enriched respectively by high VEGF-A concentration and 8Br-cAMP, low concentration of VEGF-A, and VEGF-C or angiopoietin-1. Particularly, arterial hiPSC-ECs can form a more extensive capillary network *in vivo* [28]. In general, though hiPSC-ECs provide a novel notion, their scientific and therapeutic potential still remain to be carefully assessed in clinical application (Figure 6).

Furthermore, the transplantation of EPCs, together with other factors, may be more favorable since the endothelial differentiation of CD34+ cells in co-culture with CD34 + −derived ECs may decrease the inflammatory reaction and increase the neovascularization of ischemic tissue [91]. It has been reported that the NSCs and EPCs can ultimately promote each other by migrating to the injured sites following ischemic stroke and inducing the neurogenesis and vasculogenesis of the local microcirculation [92]. An additional study showed that the transplantation of both BMSCs and EPCs provides an improvement in function, an increase in microvessel density, and a decrease in apoptotic cells and infarct volume of the ischemic boundary area [93]. Inducing bone marrow-derived angiogenesis with the treatment of G-CSF/SCF may improve neuronal survival and functional outcome [17,94]. However, co-culture of EPCs with vascular SMCs increases the expression of the mesenchymal cell markers such as alpha-smooth muscle actin, sm22-alpha, and myocardin, as well as decreases the expression of CD31 on ECs by means of an endothelial-to-mesenchymal transition (EnMT)-like process [8].

Without doubt, the cultured quality of EPCs *in vitro* cannot be ignored. Recently, Masuda et al. [95] investigated the therapeutic potential of peripheral blood mononuclear cells (PBMNCs), based on the quality and quantity (QQ) culture of EPCs, and showed that the efficacy of QQMNCs is superior to that of PBMNCs, early EPCs, and CD34+ cells mobilized by G-CSF, regardless of the methodology used, which included intramuscular transplantation, the blood perfusion recovery, histological evaluations, or quantitative reverse transcription and polymerase chain reaction assays in ischemic hind-limbs.

### Disadvantages

Nonetheless, as predicted, EPC-based treatment also contains negative aspects. To begin with, therapeutic strategies may be relatively difficult to administer. Recent studies have shown that, compared with the controls, injected bone marrow mononuclear cells after an induced hind-limb ischemia in ApoE−/− mice not only display an increased neovascularization in ischemic regions but also promote atherosclerotic plaque formation and enhance injury to the area at the same time. In addition, EPC-treated mice also display accelerated atherosclerosis along with reduced plaque stability [8]. Moreover, an excessive treatment based on EPCs may lead to abnormal intimal hyperplasia. Decano et al. [96] reported that pro-angiogenic genes not only enhance intimal hyperplasia but are also involved in the predisposition of age-associated intimal hyperplasia, which may lead to vascular stenosis and then a vicious downward spiral. Furthermore, the isolation process of autologous EPCs inadvertently increases the burden of patients who will be treated with EPCs, in terms of both medical invasiveness and financial costs [95]. However, Wang et al. [97] have suggested that Ang-1-EPCs can express lower levels of proliferating cell nuclear antigen and can inhibit neointimal hyperplasia, compared to controls. Therefore, an appropriate evaluation of the pros and cons of EPC-based therapy still remains unknown.

In conclusion, the stimulation of EPCs opens up a wide field of cell-based angiogenic therapy for ischemic stroke, whereas, it still requires further studies in order to identify the type of EPCs with the greatest therapeutic potential to be able to then formulate a detailed treatment plan.

### The regulation of inflammatory factors in improving the function and survival of EPCs

#### Pathways of regulation

**Figure 7:**
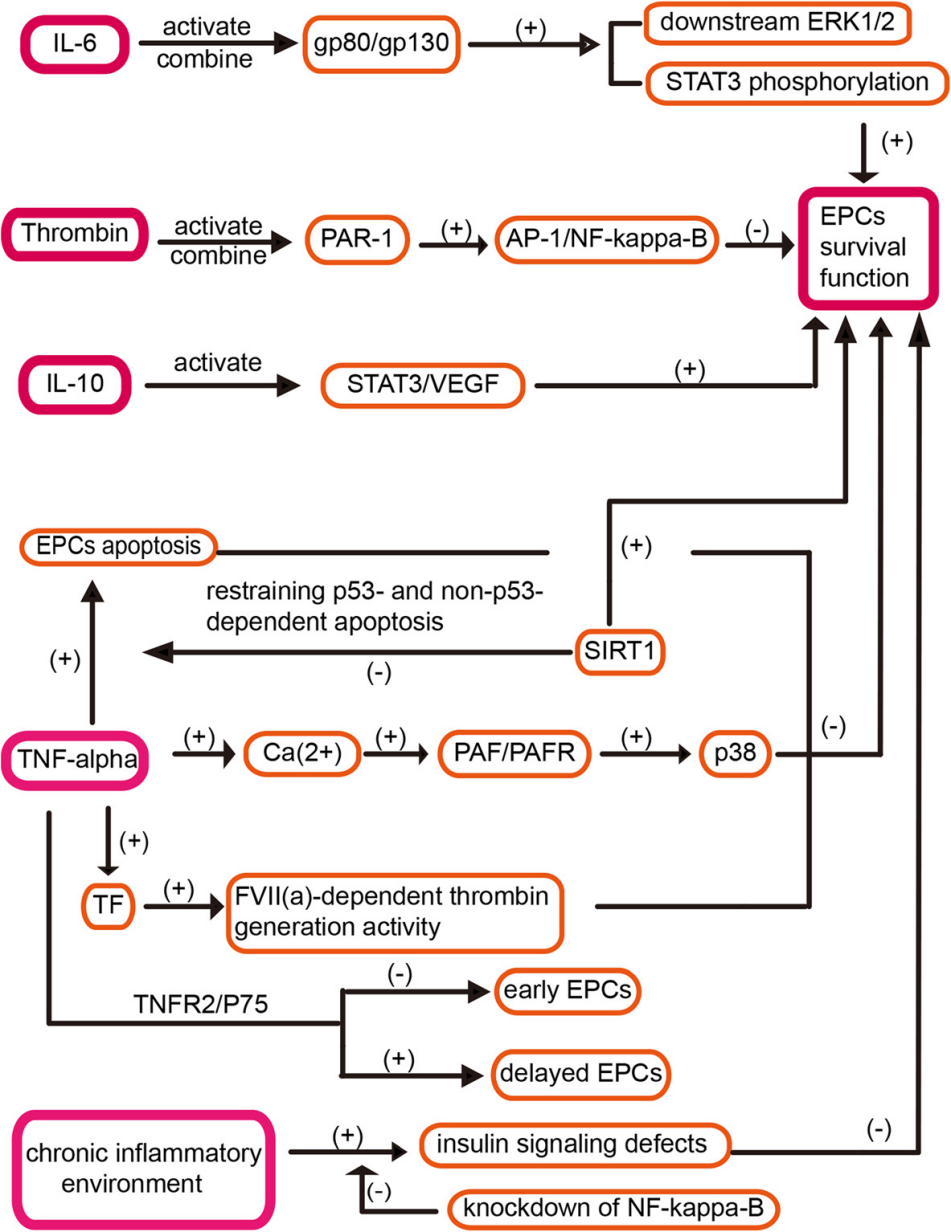
Inflammatory pathways can be regulated as therapeutic approaches for improving the function and survival of EPCs.

Although many studies have shown that EPCs have a therapeutic effect on ischemic stroke, a major challenge in translating this promise into clinical reality is our ability to efficiently deliver EPCs to target tissues and maintain a high viability and functionality. Still, the release of inflammatory factors may compromise the therapeutic efficacy; therefore, the regulation of these factors is important. Specific regulating pathways are as follows (Figure 7).

Firstly, Fan et al. [98] found that IL-6, whose receptor (gp-80) is expressed in EPCs, stimulates EPC proliferation, migration, and matrigel tube formation in a dose-dependent manner. In addition, the administration of IL-6 can activate receptor gp80/gp130 signaling pathways including downstream ERK-1/2 and signal transducer and activator of transcription-3 (STAT3) phosphorylation in EPCs. Thrombin is also a potent inflammatory factor, which can combine the functional PAR-1 that is a thrombin receptor, expressed on EPCs. What is more, Smadja et al. [99] showed that activated PAR-1 induces IL-8 synthesis from late EPCs, which involves activating protein-1 (AP-1) and NF-kappa-B pathways. Besides, Balestrieri et al. [100] unveiled a new inflammatory pathway indicating that early EPCs can express platelet-activating factor receptor (PAFR) and respond to PAF via the signaling of a transient increase of cytoplasmic Ca^2+^ concentration, which can be stimulated by TNF-alpha or high-glucose levels. PAF (more than 50 ng/ml) can reduce levels of EPCs with an increase of p38 activity, which can be abolished by a PAF receptor antagonist such as CV3988. Moreover, Cuccuini et al. [101] showed us that EPC-based therapy may be associated with prothrombotic risk. In proinflammatory conditions, tissue factors (TFs), which can trigger coagulation in ECFCs, are up-regulated in response to TNF-alpha, which confers to ECFCs a recombinant factor VII(a)-dependent thrombin generation activity without influencing their non-coagulant properties. Krishnamurthy et al. [21] indicated that mobilization of EPCs following MI is impaired in IL-10 KO mice; conversely, IL-10 increases the survival and function of EPCs, possibly through activating STAT3/VEGF signaling pathways, to further weaken MI-induced left ventricular dysfunction and remodeling. However, whether the above process occurs in ischemic stroke has yet to be explored. Furthermore, Desouza et al. [102] thought that a chronic inflammatory environment can cause insulin signaling defects in EPCs to reduce their survival, which can be reversed by modifying EPCs with a knockdown of NF-kappa-B. Additionally, modifying EPCs also decreases neointimal hyperplasia in Zucker fatty rats. This evidence suggests that inflammatory and oxidative changes can increase apoptosis of EPCs, of which TNF-alpha plays a key role as an inflammatory mediator and an inducer of apoptosis of EPCs. Additionally, silent information regulator type-1 (SIRT1), one of the sirtuin NAD-dependent protein/histone deacetylase family, can promote the survival of EPCs by restraining p53- and non-p53-dependent apoptosis in response to DNA damage and oxidative stress [103]. Lastly, Sasi et al. [104] reported that TNF-TNFR2/P75 signaling inhibits early but increases delayed non-targeted effects in bone marrow-derived EPCs.

#### Reduced sensitivity of EPCs to inflammatory activation

Previous studies have shown that, in the early stages, inflammatory factors can stimulate the activities of EPCs, but a chronic inflammatory environment is harmful to the survival and function of EPCs. Therefore, it is significant to reduce the sensitivity of EPCs to chronic inflammatory activation.

As it has been established, TNF-alpha and IL-1-beta are harmful to early EPCs, but Henrich et al. [105] elucidated that simvastatin, a 3-hydroxy-3-methylglutaryl-coenzyme-A reductase inhibitor, can protect EPCs from TNF-alpha-mediated and eventually from IL-1-beta-mediated apoptosis, which suggests that in a hyperinflammatory situation, simvastatin has protective effects on the survival of EPCs. Another report posited that all EPC subtypes release chemokines and thromboinflammatory mediators, which can be stimulated with TNF-alpha. Early EPCs primarily release thromboinflammatory mediators such as TF, but adult, late EPCs primarily release chemokines such as monocyte chemoattractant protein-1, which can be dramatically reduced by simvastatin. Zhang et al. [106] put forward that pharmacological modulation of EPCs before and after transplantation may benefit EPCs with regard to both function and survival. Liu et al. [107] presented a novel idea to make EPCs less sensitive to inflammatory stimuli, that before therapy of ischemic diseases with EPCs, EPCs from human umbilical cord blood are transduced with a lentiviral vector for stable expression of A20, an anti-inflammatory protein. Finally, Wang et al. [97] have suggested that Ang-1-EPCs can alleviate inflammatory responses induced by TNF-alpha *in vitro*, improve cell survival, and promote the endothelialization of damaged blood vessels.

**Figure 8:**
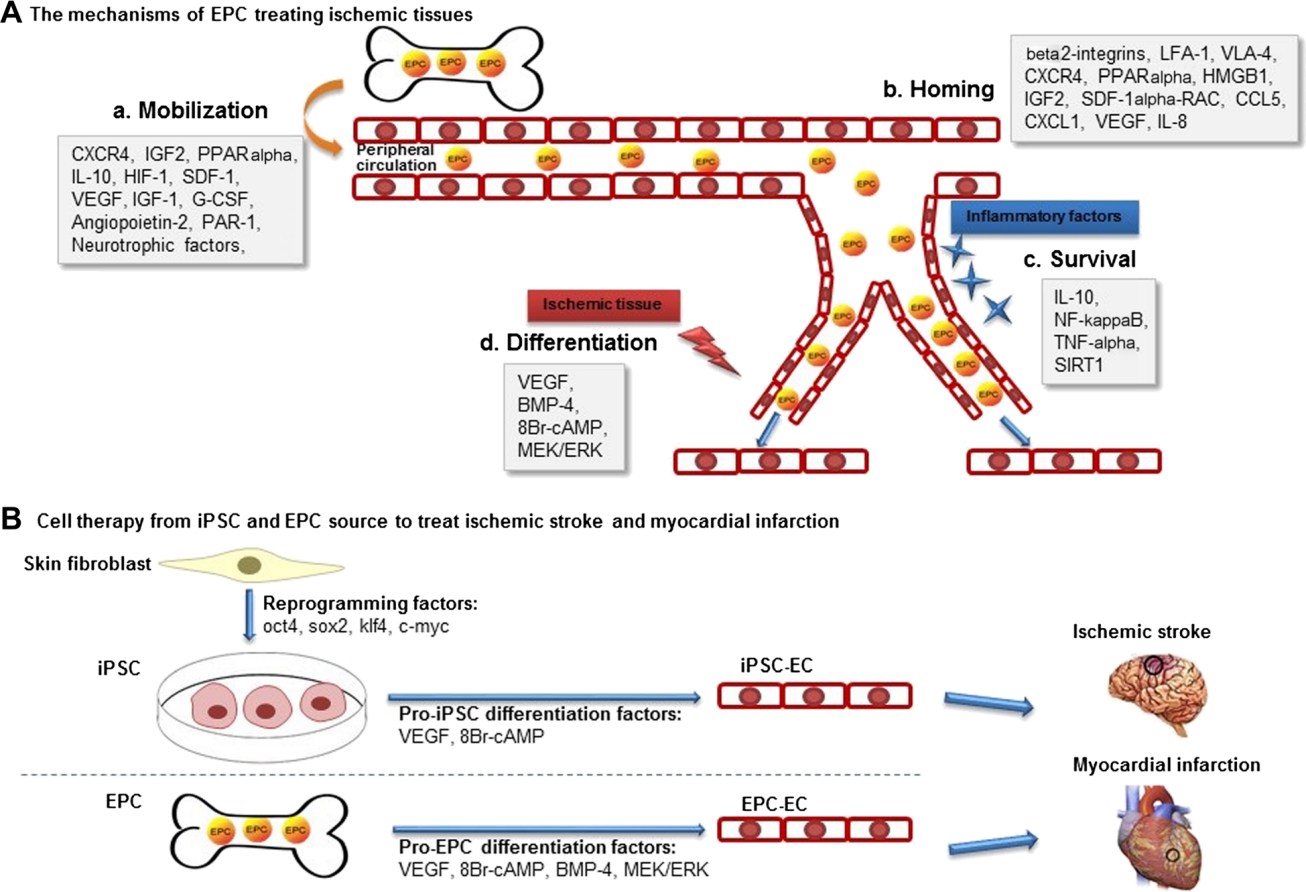
Novel EPC-based cell therapies are under development for ischemic stroke and myocardial infarction. (**A**)The mechanisms of EPCs treating ischemic tissues, including the processes of a. mobilization, b. homing, c. survival, and d. differentiation. (**B**) Cell therapy from iPSCs and EPCs source to treat ischemic stroke and myocardial infarction.

To sum up, it is a great pleasure for us to see that studies investigating the interactions between inflammatory mediators and EPCs put forward novel approaches to the therapy of ischemic stroke (Figure 8). However, the role of other inflammatory factors on EPCs is still unclear. In addition, the inflammatory stimuli have varying influences on EPCs in different periods; therefore, determining specifically when and how to intervene with regard to inflammatory stimuli still remains to be explored.

## Conclusions

As far as the role of EPCs on ischemic stroke is concerned, we have analyzed extensively the progress and existing problems in the related literature. Although it has yet to be fully determined, the focus on immune phenotypes of EPCs in ischemic stroke may enlighten us by determining whether the combination of activating different immune phenotypes can enhance EPCs’ impact on ischemic stroke. Moreover, the time distribution and quantitation of EPCs in ischemic stroke remain to be perfected and standardized. Excitedly, our extensive discussions on EPC therapy provide significant evidence to make a novel hypothesis that the inhibition of apoptosis factors and activation of the protection factors may be administrated in order to reduce negative-factor-mediated EPC toxicities or enhance EPC levels for improving EPC therapy. However, the mechanisms pertaining to mobilization, migration, and endogenous repair of EPCs after ischemic stroke need to be further explored and validated, particularly the relationship between angiogenesis and neurogenesis. Ultimately, this ambiguity is in result of the relative lack of studies done on EPC-based gene regulation, therapy, and the coinciding disadvantages. In conclusion, the role of EPCs on ischemic stroke remains to be further determined.

## Abbreviations

EPCs: endothelial progenitor cells
ECs: endothelial cells
VEGFR-2: vascular endothelial growth factor receptor-2
ECFCs: endothelial colony-forming cells
SDF-1: stromal-derived factor
G-CSF: granulocyte-colony-stimulating factor
IL-6: interleukin-6
eNOS: endothelial nitric oxide synthase
CXCR4: C-X-C chemokine receptor type 4
SCF: stem cell factor
NO: nitric oxide
IGFBP-3: insulin-like growth factor binding protein-3
MMP-9: matrix metalloproteinase-9
IGF2: insulin-like growth factor 2
HIF: hypoxia-inducible factor
PAR-1: protease-activated receptor-1
BMP4: bone morphogenic protein 4
8-Br-cAMP: 8-bromoadenosine 3′,5′-cyclic monophosphate
MAPKK: mitogen-activated protein kinase kinase
ERK: extracellular signal-regulated kinase
PDL: the periodontal ligament
LFA-1: leukocyte function-associated antigen-1
VLA-4: very late antigen-4
ICAM-1: intercellular adhesion molecule-1
VCAM-1: vascular cell adhesion molecule-1
PSGL-1: P-selectin glycoprotein ligand-1
HMGB1: high-mobility group protein B1
RAGE: receptor for advanced glycation end products
TLR2: toll-like receptor 2
SMS2: sphingomyelin synthase 2
CACs: circulating angiogenic cells
CFU: colony-forming unit
PPAR: peroxisome proliferator-activated receptor
CCL5: chemokine (C-C motif) ligand 5
CCR5: C-C chemokine receptor type 5
CXCR2: C-X-C chemokine receptor type 2
SMCs: smooth muscle cells
CXCL7: chemokine (C-X-C motif) ligand 7
DPP-IV: dipeptidyl peptidase IV
MPs: magnetic bionanoparticles
PIGF: placental growth factor
COX-2: cyclooxygenase 2
PGI2: prostacyclin
vWF: Von Willebrand factor
MCAO: middle cerebral artery occlusion
ET: endothelin
TNF: tumor necrosis factor
TIMP-1: tissue inhibitor of matrix metalloproteinases-1
EPO: erythropoietin
ACE2: angiotensin-converting enzyme 2
Ang I: angiotensin I
NOX: nicotinamide adenine dinucleotide phosphate oxidase
NF: nuclear factor
MDA: malondialdehyde
SOD: superoxide dismutase
GSH-PX: glutathione peroxidase
BNP: B-type natriuretic peptide
PKA: protein kinase A
CDP: cytidine diphosphate-choline
Alde-Low: low aldehyde dehydrogenase activity
OECs: colony-outgrowth endothelial cells
hiPSCs: human-induced pluripotent stem cells
PiPS: partial iPS
OSKM: Oct4, Sox2, Klf4, and c-Myc
OSLN: Oct4, Sox2, Lin28, and Nanog
Flk1: fetal liver kinase-1
Isl1: islet-1
bFGF: basic fibroblast growth factor
DKK1: Dickkopf homolog 1
NSC: neural stem cells
NCSC: neural crest stem cells
NGF: neural growth factor
VSMCs: vascular smooth muscle cells
EnMT: endothelial-to-mesenchymal transition
PBMNCs: peripheral blood mononuclear cells
STAT3: signal transducer and activator of transcription-3
AP-1: activating protein-1
PAFR: platelet-activating factor receptor
TF: tissue factors
MI: myocardial infarction
SIRT1: silent information regulator type-1
